# Bioinformatics prediction of miR-30a targets and its inhibition of cell proliferation of osteosarcoma by up-regulating the expression of PTEN

**DOI:** 10.1186/s12920-017-0300-3

**Published:** 2017-11-15

**Authors:** Biao Zhong, Shang Guo, Wei Zhang, Chi Zhang, Yukai Wang, Changqing Zhang

**Affiliations:** Department of Orthopedics, Shanghai Jiao Tong University Affiliated Sixth People’s Hospital, Shanghai, 200233 China

**Keywords:** MiR-30a, PTEN, Osteosarcoma, Anti-tumor

## Abstract

**Background:**

MiRNAs are frequently abnormally expressed in the progression of human osteosarcoma. Phosphatase and tensin homologue deleted on chromosome 10 (PTEN) is one of the tumor suppressors in various types of human cancer. In the present study, we detected how hsa-miR-30a-3p regulated PTEN and further tested the role of hsa-miR-30a-3p in the cell proliferation of osteosarcoma cells.

**Methods:**

The levels of miR-30a were determined by real time PCR. The expression of PTEN was tested by western blotting analysis. Cell distribution of PTEN was observed with confocal laser scanning microscope. Cell viability was determined by MTT assay.

**Results:**

The expression of miR-30a and PTEN was obviously decreased in MG-63, 143B and Saos-2 cells compared with primary osteoblasts. TargetScan analysis data showed miR-30a might bind with position 30-57 of 3’UTR of PTEN. Transfection with miR-30a-3p increased the level of PTEN in MG-63 cells, while transfection with miR-30a-3p inhibitor significantly decreased the expression of PTEN in osteosarcoma cells. Transfection with miR-30a-3p significantly inhibited cell proliferation of osteosarcoma cells, while miR-30a inhibitor obviously promoted cell viability of MG63 cells and Saos-2 cells. Inhibition of PTEN eliminated the proliferation inhibitory effect of miR-30a-3p.

**Conclusion:**

Thus, all these findings revealed the anti-tumor effects of miR-30a in human osteosarcoma cells, which could be mediated by regulating the level of PTEN.

## Background

Osteosarcoma is one of lethal diseases with highly aggressive progression and poor prognosis, which seriously threatens the health of children and young people. MicroRNAs (miRNAs) are an abundant class of evolutionarily conserved, small, single-stranded noncoding RNAs found in diverse organisms. Although the biological functions of most miRNAs are not yet fully understood, they may have a key role in the regulation of various biological processes [1]. The miRNAs have rapidly gained traction in various human diseases such as cancer, heart diseases, immune-related diseases and diabetes, etc. It has been found that miRNAs are widely involved in tumorigenesis, invasion and metastasis of osteosarcoma, in which miRNAs act as tumor suppressors or oncogenes [2]. Researches on high-throughput RNA-sequencing data revealed that miRNAs was abnormally expressed in small cell osteosarcoma specimens compared with healthy individuals, in which 37 miRNAs were dysregulated consisted of 27 up-regulated miRNAs and 10 down-regulated miRNAs [3]. The identification and expression of miRNAs in osteosarcoma patients may be reliable diagnostic and prognostic markers in the therapy of osteosarcoma [4]. Recently, more and more miRNAs were reported to play the important role in the proliferation and invasion of human osteosarcoma cells. For example, miR-543 was significantly upregulated whereas the levels of PRMT9 were obviously decreased in osteosarcoma tissues compared to the paired normal bone tissues. The data showed that miR-543 promoted cell growth in vitro and in vivo by suppressing PRMT9-enhanced cell oxidative phosphorylation, which target the 3′-UTR of PRMT9 mRNA to inhibit its translation [5]. The levels of miR-106b were significantly higher in osteosarcoma, which functioned as an oncogene to promote the progression of osteosarcoma [6]. Moreover, miR-1247 was detected to work as a potential tumor suppressor by targeting MAP3K9 in progression of osteosarcoma [7].

MiR-30a has been found to act as a tumor suppressor in various human cancers. Liu X et al. reported that miR-30a inhibited tumor growth by double-targeting COX-2 and BCL-9 in H.pylori gastric cancer models [8]. It also suppressed the progression of glioma by repression of Wnt5a, as well as the stem cell like properties [9]. In breast cancer cells, miR-30a attenuated the progression of breast cancer by down-regulating the downstream target gene, Notch1 [10]. MiR-30a also targeted the DNA replication protein RPA1 to suppress the replication of DNA and ultimately to initiate cancer cell apoptosis in gastric cancer cell models [11]. Moreover, in colon carcinoma, restoring miR-30a function suppressed tumor growth by targeting the 3′ UTR of denticleless protein homolog (DTL), which prove useful as an effective therapeutic strategy for colon carcinoma [12].

However, the role of miR-30a was not clearly clarified in human osteosarcoma. There was only one paper on miR-30a in osteosarcoma and it has reported that overexpression of miR-30a decreased the proliferation, migration and invasion of osteosarcoma cells by targeting and regulating the expression of runt-related transcription factors 2 (Runx2) [13]. In the present study, we used bioinformatics prediction software (TargetScan online analysis) to investigate the possible target gene of miR-30a in humans and the results demonstrated that miR-30a might target the 3’UTR of PTEN in human cells. Thus, we designed several experiments to investigate the role of miR-30a in the progression of osteosarcoma and explored whether PTEN was regulated by miR-30a in osteosarcoma cancer cells. The study would give new clues to clearly reveal the tumorigenesis of osteosarcoma.

## Methods

### Agents and cell lines

The human osteosacoma cancer cell lines MG63, 143B and Saos-2 were purchased from Shanghai Institute of Cell Biology, Chinese Academy of Sciences. Human primary osteoblasts (Cat.No.GN-H109) was obtained from Gaining biological corporation (Shanghai, China). The cells were maintained and cultured in DMEM medium supplemented with 10% FBS, streptomycin and penicillin (1×) in cell incubator under the humid air atmosphere with 5% CO_2_ at 37 °C. MISSION® microRNA Mimic hsa-miR-30a-3p (Cat.No.HMI0455) and negative control (Cat. No.HMC002) was purchased from Sigma. hsa-miR-30a-3p miRNA Inhibitor (Cat. No. MIH01690) and miRNA Inhibitor Negative Control (Cat.No.MIH00000) were obtained from abm corporation. PTEN inhibitor SF1670 (Cat.No.SML0684) was purchased from Sigma.

### Cell transfection

The osteosarcoma cells were transfected with miR-30a-3p mimic, negative control mimic, miR-30a-3p inhibitor and negative control inhibitor using lipofectamine 2000 transfection reagents (Invitrogen, Carlsbad, CA, U.S.A) cultured for 24, 48 and 72 h respectively according to the manual.

### MTT assay

MG63 cells and Saos-2 cells were transfected with miR-30a-3p mimic, negative control mimic, miR-30a-3p inhibitor and negative control inhibitor for the indicated period of time. Cell viability was determined by MTT assay as described in [14, 15].

### RNA isolation and qRT-PCR

Total RNA from cultured MG63 cells, 143B and Saos-2 cells was extracted with an RNApure kit (Bioteke, Beijing, China) according to the manufacturer’s instructions. Total cDNA was reverse transcribed by using PrimeScript RT reagent Kit (Takara, Dalian, China) and miRNA cDNA was reverse transcribed by one step PrimeScript miRNA cDNA Synthesis Kit (Takara, Dalian, China). MystiCq® microRNA qPCR Assay Primer hsa-miR-30a-3p (Cat. No. MIRAP00080) was purchased from Sigma. The miRNA levels were detected by qPCR with the ABI 7500 FAST real-time PCR System (Applied Biosystems, Carlsbad, USA) using SYBR Green (Takara, Dalian, China). The reference gene U6 was used in the experiment.

### Immunofluorescence

The osteosarcoma cells were plated into 6-well plate and transfected with hsa-mir-30a mimic and negative control mimic for 24 h. The cells were fixed by 4% paraformaldehyde for 10 min and permeabilized with 0.1% TritonX-100 for 5 min. Next, the cells were blocked with 2% BSA for 1 h to block the non-specific protein. Then the cells were incubated with anti-PTEN antibody (Cat. No. ab79156) overnight at 4 °C. The secondary antibody was Alexa Fluor® 488 goat anti-mouse IgG (H + L) (Cat. No. ab150113) and the commonly used concentration was 2 μg/ml for 1 h. All the antibodies were obtained from Abcam corporation. Cell nucleus was stained by DAPI at a final concentration of 1.43 μM.

### Western blotting analysis

The levels of PTEN in MG63 cells, 143B cells, Saos-2 cells and primary osteoblasts cells were determined by western blotting analysis as described in the papers [16–18]. Anti-PTEN antibody [A2b1] (Cat. No. ab79156) was a mouse monoclonal antibody [A2b1] to PTEN and purchased from Abcam corporation. Beta-actin antibody and the horseradish peroxidase-conjugated goat anti-mouse were all purchased from Santa Cruz biothechnology incoporation (Amersham).

### Statistical analysis

The data was analyzed by SPSS 20.0 software. All the data were repeated three times. The data were shown as the mean ± standard error. *p < 0.05, **p < 0.01, compared with negative control group.

## Results

**Fig. 1:**
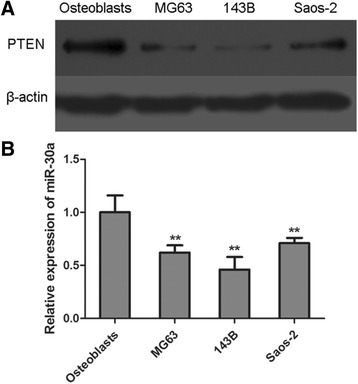
The level of PTEN and miR-30a is down-regulated in osteosarcoma cell lines.Transfection with miR-30a mimic or miR-30a inhibitor changes the relative level of miR-30a in MG63 cells. The osteosarcoma cells (MG63, 143B and Saos-2) were plated into a 48-well plate and cultured for 8 h. Primary osteoblasts were used as normal controls. **a** The levels of PTEN was determined by western blotting analysis. **b** The levels of relative expression of miR-30a was determined by real-time PCR assay. **p < 0.01, compared with osteoblasts

Firstly, we compared the expression level of PTEN in osteosarcoma cell lines (MG63, 143B and Saos-2) and normal osteoblasts. The levels of PTEN were detected by western blotting analysis and the results demonstrated that the expression of PTEN was obviously decreased in MG63, 143B and Saos-2 cell line compared with that in primary osteoblasts (Fig. 1a). Moreover, the levels of miR-30a were also determined by real time PCR. As shown in Fig. 1b, the cell lines MG63, 143B and Saos-2 showed higher level of miR-30a than that of osteoblasts (**p < 0.01, compared with osteoblasts).

**Fig. 2:**
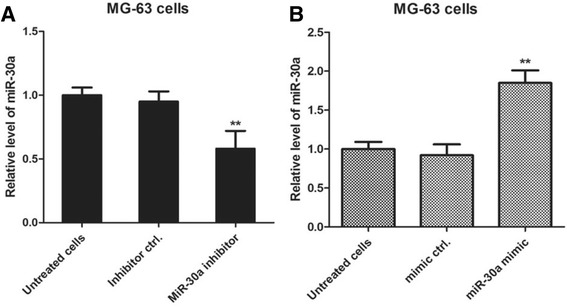
miR-30a mimic and miR-30a inhibitor is used to change the endogenous level of miR-30a. **a** The osteosarcoma cells MG63 were plated into a 48-well plate and transfected with miR-30a inhibitor and inhibitor negative control for 24 h. The relative level of miR-30a was determined by real-time PCR assay. **p < 0.01, compared with inhibitor control transfected group. **b** MG63 cells were transfected with miR-30a mimic and mimic control for 24 h and the relative level of miR-30a was determined by real time PCR assay. The relative level of miR-30a in miR-30 mimic transfected group and mimic negative control group had a significant difference (**p < 0.01)

In order to test the role of miR-30a in human osteosarcoma cell lines, MG63 cells were transfected with miR-30a inhibitor and inhibitor negative control and cultured for 24 h. The results demonstrated that the relative level of miR-30a in miR-30a inhibitor-transfected cells was significantly decreased compared with that in inhibitor negative control-transfected MG63 cells (**p < 0,01) in Fig. 2a. Conversely, miR-30a mimic-transfected MG63 cells had significantly higher level of miR-30a than that of mimic negative control-transfected MG63 cells (**p < 0.01) in Fig. 2b. There was no statistical difference between the negative control transfected cells and untreated cells.

### Transfection with hsa-miR-30a mimic increases cytoplasmic PTEN level in MG63 cells by confocal laser scanning microscope

**Fig. 3:**
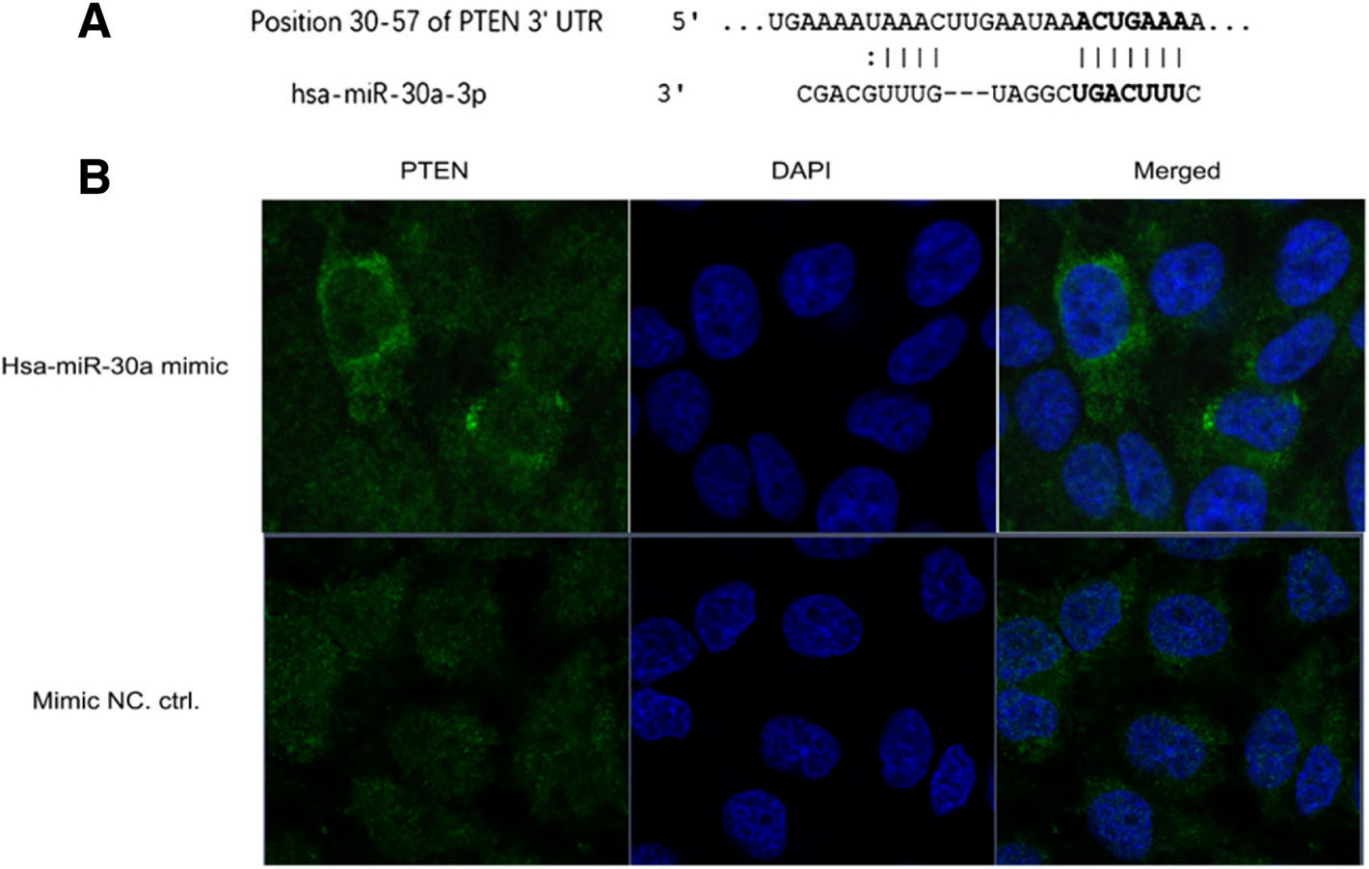
Transfection with hsa-miR-30a mimic increases PTEN expression in MG63 cells. **a** The binding sequence of miR-30a was predicted by TargetScan online ayalysis. PTEN was predicted as a target of miR-30a-3p and the position 30-57 of PTEN 3’UTR might bind with hsa-miR-30a-3p. **b** MG63 cells were transfected with hsa-miR-30a mimic and mimic negative control for 24 h. PTEN distribution was detected by immunofluorescence microscopy (200×). The staining method was described in Material and Method. DAPI was used to stain the cell nuclei at a concentration of 1.43 μM. MG63 cells transfected with mimic negative control was used as negative controls

We scanned the 3’UTR sequence of PTEN by Targetscan online analysis tool to search for probable complementary sites of human miRNAs. As shown in Fig. 3a, hsa-miR-30a-3p could bind with the position of 30-57 of PTEN 3’UTR. In order to determine the impact of hsa-miR-30a on the expression of PTEN in osteosarcoma cells, MG63 cells were transfected with miR-30a mimic and mimic negative control for 24 h and the expression of PTEN was observed by confocal laser scanning microscope. As shown in Fig. 3b, the fluorescence intensity of PTEN in miR-30a mimic transfected MG63 cells was obviously increased compared with mimic negative control transfected MG63 cells. Moreover, the fluorescence intensity was higher in cytoplasm than that of the nucleus. All the results show that overexpression of hsa-miR-30a might increase the expression of PTEN in MG63 cells and hsa-miR-30a may positively regulate the expression of PTEN.

### MiR-30a positively regulates the expression of PTEN in MG-63 cells

**Fig. 4:**
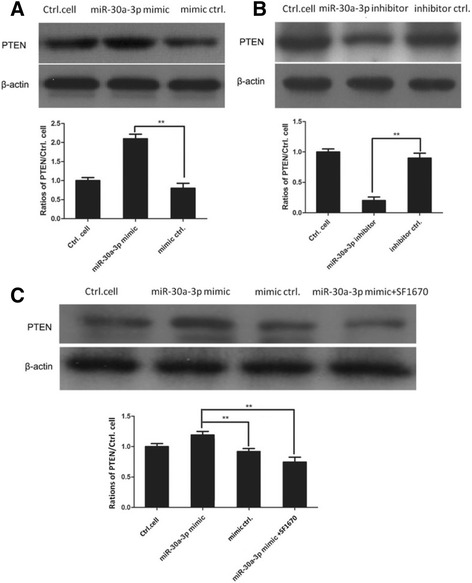
MiR-30a positively regulates the expression of PTEN in MG-63 cells. **a** MG63 cells were transfected with miR-30a-3p mimic and mimic control for 24 h. The expression levels of PTEN were determined by western blotting analysis. The gray values of PTEN were shown in histogram. **p < 0.01, compared with mimic negative control group. **b** MG63 cells were transfected with miR-30a-3p inhibitor and inhibitor negative control for 24 h. The level of PTEN was determined by western blotting analysis. There was statistical difference between miR-30a-3p inhibitor transfected cells and inhibitor negative control transfected cells (**p < 0.01). **c** MG-63 cells were pretreated with 500 nM SF1670 for 3 h and then, transfected with miR-30a-3p mimic and mimic control for 24 h. The expression levels of PTEN were determined by western blotting analysis. The gray values of PTEN were shown in histogram. **p < 0.01, compared with mimic negative control group

Next, we further explored how miR-30a regulated the expression of PTEN in osteosarcoma cells. The MG-63 cells were transfected with miR-30a-3p mimic and mimic negative control for 24 h and western blot analysis demonstrated that miR-30a-3p mimic-transfected MG63 cells had much higher level of PTEN than that of mimic negative control-transfected MG63 cells (**p < 0.01) in Fig. 4a. While the cells were transfected with miR-30a-3p inhibitor and inhibitor negative control for 24 h and the results showed that PTEN level was significantly decreased in miR-30a inhibitor transfected MG-63 cells than that in inhibitor negative control transfected MG-63 cells (**p < 0.01) in Fig. 4b. Moreover, PTEN expression was inhibited in MG-63 cells by using SF1670, a specific PTEN inhibitor which binds to the active site of PTEN. Western blot analysis suggested a significant reduction of PTEN expression in SF1670 treated cells compared with MG-63 cells transfected with miR-30a mimic only in Fig. 4c.

### MiR-30a inhibitor promotes cell proliferation of MG-63 cells and Saos-2 cells

**Fig. 5:**
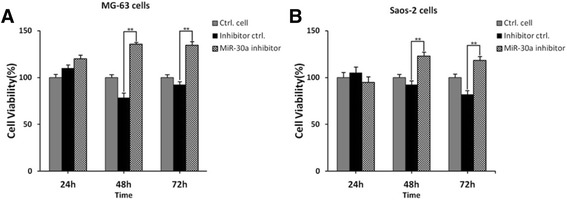
MiR-30a inhibitor promotes cell proliferation of MG-63 cells and Saos-2 cells. MG-63 cells (**a**) and Saos-2 (**b**) cells were transfected with miR-30a inhibitor and negative inhibitor control for 24, 48 and 72 h, respectively. OD490 values were determined by MTT assay. **p < 0.01, compared with negative control inhibitor-transfected cells

The functional experiment of miR-30a on the cell proliferation of osteosarcoma cells were tested by MTT assay. The MG-63 cells and Saos-2 cells were transfected with miR-30a inhibitor and inhibitor negative control for 24, 48 and 72 h, and the cell viability was determined by MTT assay. As shown in Fig. 5a, MG-63 cells transfected with miR-30 inhibitor showed higher cell viability than that of inhibitor negative control transfected MG-63 cells (**p < 0.01, compared with inhibitor negative control transfected cells). There was no statistical difference between inhibitor negative control transfected cells and untreated cells. This was consistent with the results from Saos-2 cells (Fig. 5b).

### MiR-30a mimic suppresses cell proliferation of MG-63 cells and Saos-2 cells

**Fig. 6:**
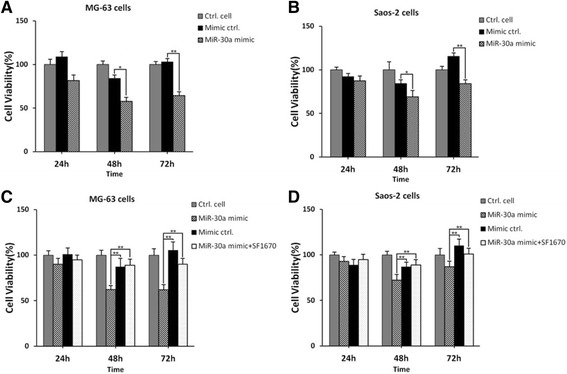
MiR-30a mimic suppresses cell proliferation of MG-63 cells and Saos-2 cells. MG-63 cells (**a**) and Saos-2 cells (**b**) were transfected with miR-30a mimic and negative control mimics. MG-63 cells (**c**) and Saos-2 cells (**d**) were pretreated with 500 nM SF1670 for 3 h and then, transfected with miR-30a-3p mimic and mimic control. The cells were cultured for 24, 48 and 72 h, respectively. Cell viability was determined by MTT assay and OD490nm values were tested. *p < 0.05, **p < 0.01, compared with negative control mimic-transfected osteosarcoma cells

Furthermore, we used miR-30a mimic and mimic negative control to transfect MG-63 cells and Saos-2 cells and the cells were cultured for 24, 48 and 72 h. As shown in Fig. 6a and b, cell proliferation value was significantly decreased in miR-30a mimic transfected MG63 cells and Saos-2 cells, compared with that in mimic negative control-transfected cells (*p < 0.05, **p < 0.01). Next, the expression of PTEN was inhibited by SF1670, as shown in Fig. 6c and d. The inhibition of PTEN expression promoted cell proliferation compared with MG-63 cells and Saos-2 cells transfected with miR-30a mimic exclusively. All the results demonstrated that transfection with miR-30a mimic could obviously suppress the cell viability of human osteosarcoma cells.

## Discussion

Osteosarcoma is the most common malignant bone tumor in children and adolescents. The profile of miRNA was abnormally expressed in the tumorigenesis and metastasis of human osteosarcoma. In the present study, we tested the expression of miR-30a in osteosarcoma cell lines, such as MG63, 143B and Saos-2 cells. We found that the level of miR-30a was obviously decreased in osteosarcoma cell lines compared with the normal control cells. Importantly, we predicted the possible binding site of the targeted gene by TargetScan online analysis, and the data showed that hsa-miR-30a might bind at the position 30-57 of 3’UTR of PTEN. This was further to be confirmed by immunofluorescence microscopy. Next, we also tested the expression of PTEN in osteosarcoma cell lines and the western blotting results showed that the expression of PTEN was obviously decreased in MG63 cells, 143B and Saos-2 cells. This suggested that miR-30a might positively regulate the level of PTEN.

Next, we used miR-30a-3p mimic and miR-30a-3p inhibitor to transfect MG63 cells. PTEN level was detected and the western blotting analysis results showed that PTEN expression was significantly decreased in miR-30a-3p mimic-transfected MG63 cells, whereas the PTEN level was obviously decreased in miR-30a-3p inhibitor transfected cells compared with that in negative control inhibitor-transfected MG63 cells. Functional experiment such as MTT assay was performed to test the role of miR-30a in the proliferation of osteosarcoma cell lines. MTT assay results revealed that transfection with miR-30a mimic inhibit cell proliferation of MG-63 cells and Saos-2 cells. The inhibition rate of miR-30a mimic in osteosarcoma cells showed a time-dependent manner. While inhibition of PTEN expression enhanced the proliferation of miR-30a-3p mimic-transfected MG63 cells and Saos-2 cells. Meanwhile, transfection with miR-30a inhibitor obviously promoted cell viabiliy of MG-63 cells and Saos-2 cells. The result was consistent with Zhang’s results, however, the difference was due to that they found miR-30a regulated cell proliferation and migration of human osteosarcoma by targeting Runx2 [12], while we found that miR-30a-3p might regulate cell proliferation by targeting the oncogene PTEN in osteosarcoma cells.

## Conclusions

In this study, we found miR-30a regulated the proliferation of human osteosarcoma cells by regulating the levels of target gene PTEN, which will give new clues in the clinical therapy of human osteosarcoma.

## Abbreviations

3’UTR: 3’Untranslated Regions
COX-2: Cyclooxygenase-2
MTT: 3-4,5-dimethyl-2-thiazolyl)-2,5-diphenyl-2-H-tetrazolium bromide
PCR: Polymerase Chain Reaction
PRMT9: Protein arginine methyltransferases 9
PTEN: Phosphatase and tensin homologue deleted on chromosome 10
Runx2: Runt-related transcription factor 2
